# RAS: Striking at the Core of the Oncogenic Circuitry

**DOI:** 10.3389/fonc.2019.00965

**Published:** 2019-09-24

**Authors:** Ryan C. Gimple, Xiuxing Wang

**Affiliations:** ^1^Division of Regenerative Medicine, Department of Medicine, University of California, San Diego, San Diego, CA, United States; ^2^Department of Pathology, Case Western University, Cleveland, OH, United States; ^3^Key Laboratory of Antibody Technique of Ministry of Health, Nanjing Medical University, Nanjing, China

**Keywords:** RAS, cancer, metabolism, immunology, mitogen activated kinase, cancer therapy

## Abstract

Cancer is a devastating disease process that touches the lives of millions worldwide. Despite advances in our understanding of the genomic architecture of cancers and the mechanisms that underlie cancer development, a great therapeutic challenge remains. Here, we revisit the birthplace of cancer biology and review how one of the first discovered oncogenes, RAS, drives cancers in new and unexpected ways. As our understanding of oncogenic signaling has evolved, it is clear that RAS signaling is not homogenous, but activates distinct downstream effectors in different cancer types and grades. RAS signaling is tightly controlled through a series of post-transcriptional mechanisms, which are frequently distorted in the context of cancer, and establish key metabolic and immunologic states that support cancer growth, migration, survival, metastasis, and plasticity. While targeting RAS has been fiercely pursued for decades, new strategies have recently emerged with the potential for therapeutic efficacy. Thus, understanding the complexities of RAS biology may translate into improved therapies for patients with RAS-driven cancers.

## Introduction

The RAS family represents some of the earliest described oncogenes and its discovery fundamentally transformed our understanding of cancer biology. Originally identified in the 1960s as a viral component that induced formation of sarcomas in rats (1, 2), the RAS oncogenes were later found to be normal components of the human genome (3, 4) that were capable of transforming normal human cells (5, 6). Since these early studies, additional work has highlighted the importance of RAS as a contributor to many human cancers and has more fully elucidated its signaling axis and molecular regulators. As a small membrane-localized GTPase, RAS proteins integrate a number of proliferative signals to establish a tumorigenic cellular circuit when aberrantly activated. Encoded by the *KRAS4A, KRAS4B, HRAS*, and *NRAS* genes, RAS family members are among the most frequently altered oncogenes in human cancers. In this review, we dissect the oncogenic circuitry established by RAS and discuss its numerous roles in supporting proliferative signaling, survival pathways, metabolic and immunologic functions, and its potential vulnerabilities as a therapeutic target.

## RAS Signaling Cascade and Regulation

RAS signaling can be activated by a number of cellular receptors including receptor tyrosine kinases (RTKs), G-protein coupled receptors (GPCRs), and integrin family members. These signaling cascades initiate RAS activation through assembly of several scaffolding proteins that mediate conversion of RAS from an inactive GDP-bound form to an active GTP-bound state. Epidermal growth factor receptor (EGFR) is a member of the RTK family and one of the best characterized activators of RAS signaling through recruitment of the molecular scaffolding protein growth factor receptor bound protein 2 (GRB2) (7). GRB2 recruits the RAS-guanine exchange factor (RAS-GEF) SOS1, which activates the RAS protein through a conformational change induced by exchanging GDP for GTP. Similarly, other RTK family members including platelet derived growth factor receptor beta (PDGFR-β) can initiate RAS activation through recruitment of GRB2 (8), and colony stimulating factor 1 receptor (CSF-1R) signaling functions through activation of RAS (9). Several GPCRs also function in a RAS-dependent manner with the beta-gamma subunit of GPCRs activating RAS signaling (10). GPCRs activate RAS through stimulation of both non-RTKs (11) (including src, Lyn, and Syk) and RTKs as described above. Certain downstream signaling functions of integrin proteins are also RAS dependent (12).

RAS can be further activated by additional RAS-GEFs including the RAS-GRF and RAS-GRP family members or negatively modulated by a series of RAS-GTPase activating enzymes (RAS-GAPs), including neurofibromin 1 (NF1) (13). These RAS activity regulators are also frequently altered across a number of cancer types. Post-translational modifications are also critical to the functions of the RAS protein. The addition of an isoprenyl group (farnesylation) by farnesyl transferase is essential for RAS localization to the plasma membrane and downstream signaling roles (14). Further, palmitoylation of the NRAS and HRAS proteins by the enzymes DHHC9 and GCP16 promotes membrane localization and efficient signaling (15). Continuous cycles of NRAS and HRAS palmitoylation ensure that these proteins are selectively localized to the Golgi or plasma membrane and not in other intracellular membranes (16, 17). KRAS, however, can localize to the plasma membrane without the requirement of palmitoylation (18). The post-translational membrane anchor that fastens KRAS to the plasma membrane contains unique sequences and electrostatic properties that determine the specific localization of RAS nanoclustering within anionic phospholipids (19). KRAS dimerization is also critical for oncogenic signaling (20).

**Figure 1:**
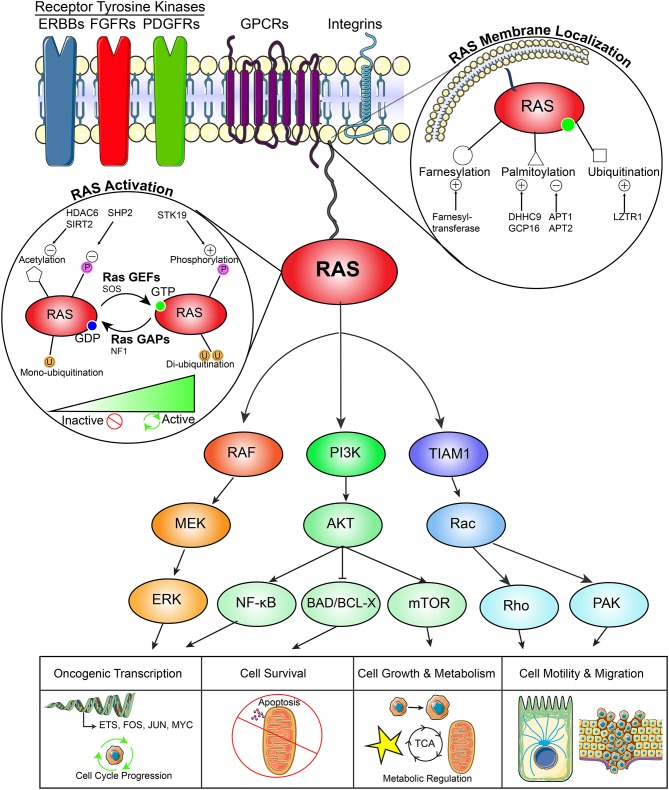
RAS pathway in cancer. This diagram demonstrates (1) the upstream activators of RAS signaling (2) regulators of RAS membrane localization, (3) regulators of RAS activity, (4) downstream signaling effector pathways, and (5) downstream functional effects of RAS signaling in cancers.

Further post-translational modifications including mono-ubiquitination favor the active form of RAS (21, 22), while di-ubiquitination decreases downstream signaling output through ERK (23). RAS signaling can be abrogated through ubiquitination by an LZTR1-CUL3 complex, which inhibits its membrane localization (24, 25). RAS acetylation has also been shown to reduce signaling activity, with cells dependent on the protein deacetylases HDAC6 and SIRT2 to maintain RAS signaling (26, 27). Additionally, acylpeptide hydrolase (APEH) contributes to the appropriate localization of RAS to the plasma membrane by regulating phosphatidylserines in the plasma membrane (28) (Figure 1).

Following activation, RAS can execute a variety of functions that promote cancer development including oncogenic transcription, cell cycle progression, cellular survival, cell growth and metabolism, and cell motility and migration. First, RAS activates the mitogen-activated protein kinase (MAPK) pathway defined by a RAF-MEK-ERK signaling axis. This pathway activates transcription of a number of proliferative signaling networks driven by FOS, JUN, and ETS family transcription factors, as well as MYC. These factors support cancer cell proliferation through promoting cell cycle entry, angiogenesis, and survival. Second, RAS plays an important role in the activation of the PI3K-AKT signaling network, which supports oncogenic transcription through NF-κB signaling, evasion of apoptosis through inhibition of the pro-apoptotic enzyme BAD, and cell growth and metabolism through mTOR. Third, activation of TIAM1 drives cancer cell motility and migration through a Rac-Rho and Rac-PAX dependent network. Other RAS effectors have been studied extensively (29) (Figure 1).

KRAS can also mediate activation of canonical Wnt signaling while suppressing non-canonical Wnt pathways to promote tumor growth. In APC-deficient colon cancers, KRAS-dependent cells specifically upregulate BMP signaling, which activates expression of TAK1/MAP3K7 and downstream transcriptional upregulation of canonical Wnt target genes. This pathway can be targeted with TAK1 kinase inhibitors, which selectively ablate KRAS-mutant colon cancer xenografts (30). KRAS has also been shown to inhibit non-canonical Wnt signaling through sequestering calmodulin and blocking transcription of the Frizzled 8 receptor, a G protein-coupled receptor activator of non-canonical Wnt signaling (31). This represents one distinguishing feature between RAS family proteins, as HRAS is unable to similarly affect this pathway (31). Because non-canonical Wnt signaling reduces activation of canonical Wnt signaling pathways, these studies consistently show that KRAS activates canonical Wnt signaling to support stem-like properties of cancer cells and tumor growth and that this node may be targeted for cancer therapy.

## Role of RAS Mutations in Different Cancer Types

**Figure 2:**
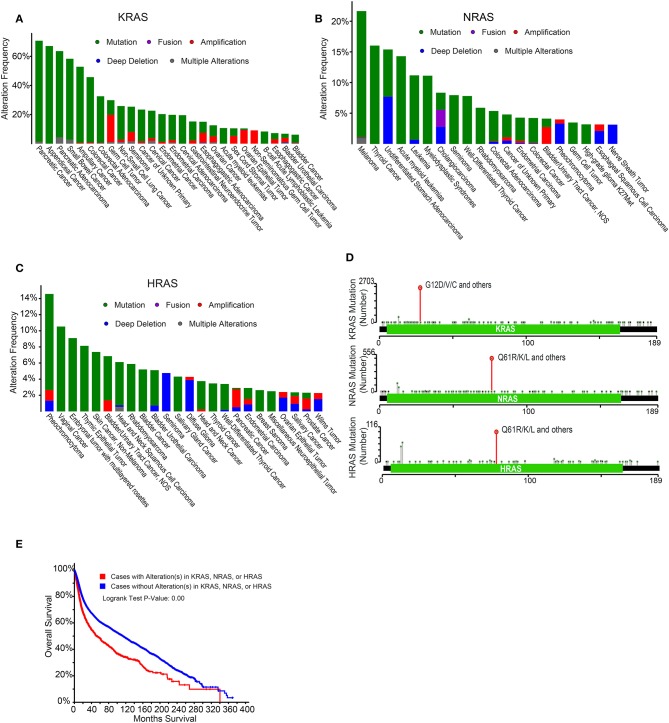
Epidemiology of RAS alterations in cancer. **(A)** Frequency of KRAS alterations across a number of cancer types. Data were derived from Cerami et al. (33) and Gao et al. (34). **(B)** Frequency of NRAS alterations across a number of cancer types. Data were derived from Cerami et al. (33) and Gao et al. (34). **(C)** Frequency of HRAS alterations across a number of cancer types. Data were derived from Cerami et al. (33) and Gao et al. (34). **(D)** Localization of RAS gene mutations across the gene body. Data were derived from Cerami et al. (33) and Gao et al. (34). **(E)** Prognosis of cancer patients with or without alterations in KRAS, NRAS, or HRAS. Data were derived from Cerami et al. (33) and Gao et al. (34).

**Figure 3:**
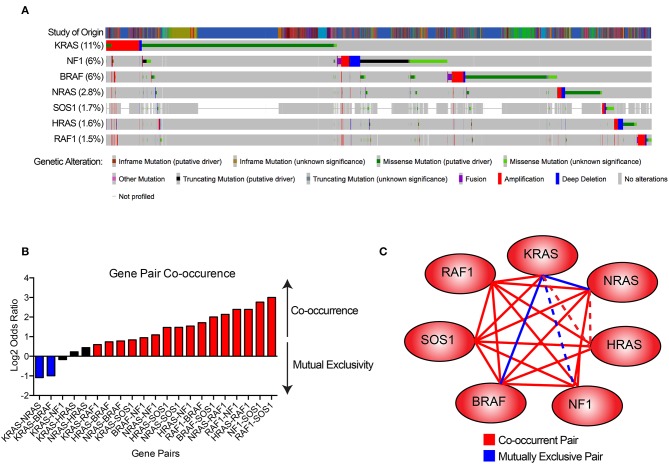
Gene pair co-occurrence among RAS pathway genes. **(A)** Co-occurrence plot RAS pathway genes across a number of cancer types. Data were derived from Cerami et al. (33) and Gao et al. (34). **(B)** Gene pair co-occurrence plot of RAS pathway genes. Blue bars indicate gene pairs that are significantly mutually exclusive, red bars indicate gene pairs that are significantly co-occurrent, and black bars indicate gene pairs without significant co-occurrence. **(C)** Gene pair co-occurrence network. Solid blue lines indicate gene pairs that are significantly mutually exclusive, solid red lines indicate gene pairs that are significantly co-occurrent, and dotted lines indicate gene pairs without significant co-occurrence.

The Cancer Genome Atlas (TCGA) project identified the RTK-RAS signaling pathway as the most frequently altered oncogenic network in cancer, with 46% of all samples displaying alterations (32). RAS alterations contribute to 20–30% of all human cancers. KRAS mutations are exceedingly common in pancreatic adenocarcinomas and colorectal cancers, while NRAS mutations are more common in melanomas, thyroid cancers, and leukemias (33, 34) (Figures 2A–C). Although KRAS, HRAS, and NRAS share functional similarities, KRAS missense gain-of-function mutations tend to occur on the 12th codon, while those in HRAS and NRAS occur on the 61st codon and are differentially utilized across cancer types (33–35) (Figure 2D). These mutations act by creating enhanced RAS activity, effectively uncoupling pro-proliferative downstream signaling from growth factor receptors. Alterations in any of these RAS family genes is associated with poor patient prognosis in pan-cancer analyses (33, 34) (Figure 2E), and RAS pathway gene alterations frequently co-occur with the exception of KRAS-BRAF and KRAS-NRAS gene pairs, which are mutually exclusive (33, 34) (Figure 3).

Pancreatic ductal adenocarcinomas (PDACs) are highly lethal and display exceptionally high frequency of KRAS mutations (94% mutant). RAS mutations in PDAC commonly co-occur with CDKN2A mutations and deletions, TP53 mutations, and SMAD4 mutations (36–38). Colorectal cancers are largely initiated by mutations in APC, which lead to uncontrolled Wnt signaling, followed by loss of function of TP53, inactivation of TGF-β signaling, and mutations in KRAS in ~37% of cases (39). KRAS is the most commonly mutated oncogene in lung adenocarcinoma, occurring in 33% of cases, along with EGFR, BRAF, and TP53 mutations (40). Despite the high prevalence of KRAS mutations and RTK activation in lung adenocarcinomas (and other forms of non-small cell lung cancers), small cell lung carcinomas are characterized by nearly universal inactivation of TP53 and RB1 through mutation or deletion, without alterations in RAS (41). In contrast to pancreatic, lung, and colon cancers, melanomas contain NRAS mutations in 20–30% of cases (42) NRAS is also commonly mutated in acute myeloid leukemias in 15% of cases (43, 44).

The differential mutation rate across cancers suggests that each mutational event may activate distinct signaling events and that each tissue type may be differentially poised to transform following RAS mutation. For example, HRAS displayed a greater capacity to transform fibroblasts than the other RAS family members (45), while in hematopoietic cell models, NRAS demonstrated a stronger transforming potential (46). RAS family members display distinct post-translational modifications, which regulate their subcellular localization and differential signaling preferences, which have been extensively reviewed elsewhere (47–49).

## RAS and Metabolism

Dysregulated metabolism is a key hallmark of cancer, and activation of RAS signaling supports cancer initiation, maintenance, and progression through driving altered metabolic networks. RAS signaling promotes oncogenic metabolism by coordinating numerous metabolic processes including lipid, nucleotide, and glycolytic pathways. Specifically, RAS signaling supports cellular bioenergetic needs and enhances glucose uptake through induction of the GLUT1 glucose transporter promoting survival in low-nutrient conditions and increased glycolytic metabolism (50). This glucose is shunted away from the tricarboxylic acid (TCA) cycle to support glycolytic metabolism, protein glycosylation, and nucleotide metabolism through the pentose phosphate pathway (51, 52). Cells also upregulate glutamine metabolism and the phosphoserine biosynthetic pathway through upregulation of biosynthetic enzymes in these pathways (53). KRAS redirects glutamine utilization to support cellular redox balance through transcriptional regulation of the GOT1 (glutamic-oxaloacetic transaminase 1) enzyme and creates a dependency on glutamine metabolism (54). Co-mutation of KRAS with loss of KEAP1 (kelch like ECH associated protein 1) further extended the glycolytic phenotype, dependence on glutamine, and sensitivity to glutaminase inhibitors in lung adenocarcinoma models (55). RAS signaling also acts to support nucleotide biosynthesis via MYC activation. RAS upregulates MAPK signaling, which induces MYC and drives nucleotide metabolism through the pentose phosphate pathway (56).

Increased copy number of mutant oncogenic KRAS that typically occurs later in the process of tumorigenesis further activates glycolytic metabolism and supports glutathione synthesis, but can also direct metabolites into the TCA cycle in lung cancer cells to support tumor progression (57). This mitochondrial metabolism has been shown to be essential for anchorage-independent cell growth in KRAS-driven cancers by promoting generation of reactive oxygen species, which modulate ERK signaling (58). This suggests that differential dosage of KRAS expression can have contrasting effects on cellular metabolism and highlights the evolution of metabolic states throughout tumor development. RAS allelic imbalance and loss of wild-type KRAS alleles can further extend the oncogenic properties of cancer cells and mark the most aggressive undifferentiated cells (59), but also create a dependency on the MAPK signaling pathway with unique sensitivities to pharmacologic MEK inhibition (60).

While cancers rely heavily on endogenous synthesis of substrates for anabolic needs, RAS-driven cancers also utilize mechanisms to recover materials from their extracellular environments in the form of micropinocytosis (61, 62). This process supports cancer cell growth through scavenging extracellular amino acids for use in protein synthesis, and glutamine for a variety of metabolic processes (63). RAS activation can also support cell membrane biosynthesis through fatty acid uptake from lysophospholipids in the surrounding microenvironment, reducing dependence on endogenous lipid synthesis (64). KRAS signaling sustains cancer cells under conditions of nutrient stress by activating an NRF2-ATF4 axis to increase amino acid transport and protein biosynthesis, preventing apoptotic cell death through increased asparagine synthase activity (65).

**Figure 4:**
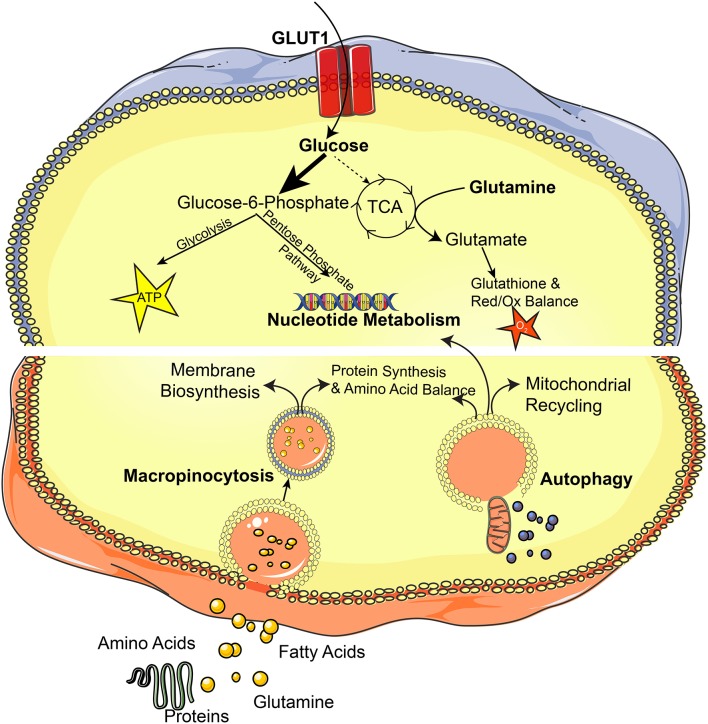
The RAS pathway orchestrates cellular metabolism. This diagram depicts metabolic pathways that are altered in RAS-driven cancers.

Despite this metabolic resiliency through increased nutrient scavenging capacity, RAS driven cancers are dependent on autophagy, which is essential for mitochondrial recycling and oxidative capacity (66). Autophagy is essential for proper mitochondrial function and nucleotide synthesis in KRAS-driven tumors (67), as well as for efficient catabolism of fatty acids (68). In RAS driven pancreatic cancers, autophagy is supported by the MiT/TFE family of transcription factors, including MITF, TFE3, and TFEB, which activate genes that promote autophagy and lysosomal pathways to maintain intracellular amino acid pools (69). The acyl-CoA synthetase family member, ACSL3, whose expression is driven by mTOR signaling downstream of RAS, specifically regulates intracellular fatty acid metabolism and utilization in RAS-dependent cancers by supporting fatty acid uptake, accumulation, and β-oxidation (70). Interestingly, RAS-driven metabolic dependencies can also be tissue- and context-dependent. Branched-chain amino acid metabolism is a key dependency in KRAS-driven non-small-cell lung carcinoma (NSCLC) cells in which they are essential for non-essential amino acid and DNA synthesis. However, these metabolic circuits are dispensable in KRAS-driven pancreatic ductal adenocarcinoma (PDAC) cells (71) (Figure 4).

## RAS in Cancer Metastasis

In addition to driving processes essential for early phases of tumorigenesis, RAS activity is important for the acquisition of more malignant features, including supporting metastasis. In mouse models of colorectal cancer, while primary tumors were characterized by a heterogeneous population of cells bearing both oncogenic KRAS mutations and wild-type KRAS, metastatic sites were largely comprised of more uniform cell populations harboring oncogenic KRAS (72). This metastatic phenotype was promoted by transforming growth factor beta (TGF-β) signaling (72). Distinct from heterogeneity in cellular populations with respect to KRAS mutation status, acquisition of multiple oncogenic KRAS mutations within single cells through focal amplifications and loss of the wild-type allele (loss of heterozygousity) can promote tumor metastasis and aggressive properties (59). KRAS also supports metastatic dissemination through repression of Raf Kinase Inhibitory Protein (RKIP), a putative tumor suppressor with roles in cell migration, motility, and epithelial-to-mesenchymal transition (73). Activation of KRAS signaling along with homozygous deletion of LKB1 (also known as STK11 or serine/threonine kinase 11) promoted cancer progression and metastasis in non-small cell lung cancer models (74). In KRAS-driven pancreatic cancer models, deletion of LKB1 enhanced the tumorigenicity and proliferation rate of cancer cells through enhanced serine biosynthesis and S-adenosyl-methionine (SAM), which supports DNA methylation (75).

## RAS and the Immune System

Interactions between cancer cells and the immune system are essential features of cancer biology. In order to survive and thrive, cancer cells must avoid immunoediting by immune effector cells; however, cancer cells also frequently gain proliferative advantage from the surrounding immune microenvironment (76). RAS signaling reduces expression of MHC class I molecules on the surface of cancer cells, rendering them less vulnerable to immune-mediated cell death by cytotoxic T-cells (77, 78). Immune checkpoints such as PD-L1 (CD274) serve to dampen the reactivity of the immune system and to prevent autoimmunity. Cancers frequently subvert this mechanism to avoid being targeted by the immune system. RAS signaling can promote this effect in an MEK-dependent manner by stabilizing PD-L1 mRNA through downregulation of tristetraprolin (TTP/ZFP36), an RNA binding protein which typically degrades mRNAs (79). These findings may partially explain the observation that KRAS mutant non-small cell lung cancer patients display better responses to PD-1 inhibition with nivolumab than KRAS wild-type patients (80, 81). In hepatocellular carcinoma models, dual KRAS and MYC signaling can translationally enhance PD-L1 levels by bypassing upstream open reading frames, which typically serve a repressive role (82). This contributes to a more aggressive and metastatic phenotype with the capacity to evade the immune system. KRAS and MYC signaling further cooperate to promote the development of aggressive and invasive adenocarcinomas by recruiting immunosuppressive macrophages via the chemokine CCL9 and excluding T-cells and NK cells via interleukin-23 (IL-23) (83). These alterations allow developing tumors to evade immune-mediated attack. In lung cancer models, KRAS supports expression of IL6-mediated chronic inflammation, which reorganizes the tumor microenvironment by recruiting myeloid derived suppressor cells (84, 85). Targeting MAPK and CDK4/6 pathways in RAS mutant lung cancer cells leads to natural-killer (NK) cell-mediated attack of tumor cells through induction of senescence pathways (86). Activation of the MEK/ERK signaling pathway by the oncogenic KRAS G12D mutation increases secretion of IL-10 and TGF-β from pancreatic cancer cells, which promotes conversion of T-cells to an immunosuppressive regulatory T-cell (Treg) state (87). Additionally, co-mutation with STK11 is associated with a reduction in NF-κB signaling in RAS mutant tumors and suppression of tumor immunosurveillance while co-mutation with TP53 is associated with increased immune responses (88). This suggests that mutations commonly co-occurring with RAS impinge upon the immune reactivity of RAS driven cancers.

**Figure 5:**
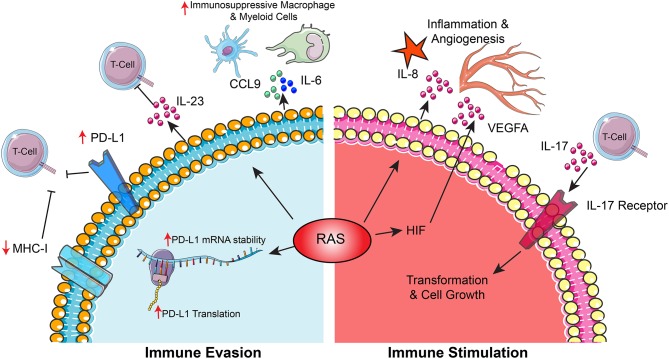
The RAS pathway shapes interactions between cancer cells and the immune microenvironment. This diagram depicts mechanisms by which RAS signaling promotes cancer through (1) supporting cancer cell immune evasion and (2) driving immune-mediated stimulation of cancer cell growth.

Besides avoiding immune-mediated destruction, cancer cells frequently benefit from a proinflammatory microenvironment that sustain oncogenic processes. In pancreatic intraepithelial neoplasia models of pancreatic cancer precursor lesions, KRAS signaling induced expression of IL-17 receptors on preneoplastic cells and infiltration by IL-17 secreting T-cells, both of which accelerated progression to a neoplastic state (89). RAS signaling also promotes tumor vascularization and inflammation by inducing secretion of IL-8 from cancer cells through MAPK and PI3K pathways (90). Tumor vascularization is further driven by KRAS-mediated induction of hypoxic HIF signaling, which drives expression of vascular endothelial growth factor (VEGF) (91). KRAS activation can activate inflammatory processes in lung cancer models by stimulating accumulation of macrophages and neutrophils through production of inflammatory chemokines (92) (Figure 5).

## Therapeutic Targeting of RAS

Because of the numerous ways in which RAS activity supports tumor cell proliferation, survival, metabolism, microenvironmental interactions, and immune evasion, efficient therapeutic targeting of RAS has been the focus of a large body of research. While it was previously believed that RAS is an undruggable target due to its molecular structure, new insights into its biological functions and molecular regulators may allow for efficient pharmacological inhibition of RAS effectors and discoveries of synthetic lethality.

### Direct RAS Inhibitors

Direct inhibition of oncogenic RAS could be a powerful therapeutic approach to ablate RAS-driven tumors. Studies of the molecular structure of the common KRAS G12C variant have informed the development of specific inhibitors that selectively target the mutant form of KRAS and both limit its activation by favoring binding to GDP as well as blocking its downstream signaling through RAF (93). Another compound targeting the KRAS G12C variant, ARS-853, selectively reduced the frequency of the active, GTP-bound KRAS, and inhibited cell proliferation in lung cancer models and suggests that nucleotide cycling between GDP and GTP bound forms are essential for its molecular functions (94, 95). Next-generation forms of KRAS G12C targeting agents, including ARS-1620, demonstrated improved potency compared to earlier generation agents and block oncogenic RAS signaling and tumor growth *in vivo* in a target-specific manner in non-small cell lung cancer models (96). These agents have been extensively reviewed elsewhere (97, 98). In addition to mutation-specific RAS inhibitors, pan-RAS inhibitors that target HRAS and NRAS as well as KRAS have been developed. One of these pan-RAS inhibitors, compound 3,144, efficiently silenced PI3K-AKT and MEK-ERK signaling downstream of RAS and prevented growth of RAS-driven xenograft cancer models. However, some off-target effects and toxicities apparent in this first-generation compound have prevented wide-spread clinical adoption at this time (99). To advance rational design of compounds with RAS targeting potential, computational modeling of RAS three-dimensional structure revealed conformational changes that occur during RAS deactivation, suggesting that stabilizing these inactive forms may reduce RAS signaling efficacy (100). Similar efforts identified a high-affinity allosteric KRAS inhibitor that impairs KRAS signaling and cancer cell growth in cells bearing several distinct types of KRAS activating mutation (101). Detailed conformational dynamics analyses and structural biology approaches uncovered numerous vulnerabilities and co-dependencies of the RAS enzyme, which may be exploited for therapeutic targeting and which have been detailed extensively elsewhere (102, 103).

In addition to small molecule inhibitors, other therapeutic approaches have investigated methods to deliver nucleic acid-based delivery of therapeutic compounds to cancer cells *in vivo*. Using nanoliposomal delivery of KRAS-targeting siRNAs, KRAS mRNA expression could be dramatically reduced with subsequent decrease in tumor growth and metastatic potential in colon and lung cancer models (104). Nanoliposomes can also be used to deliver miRNAs that specifically target KRAS and impair tumor growth and metastasis in lung cancer models (105). Cyclodextrin polymer nanoparticles can also be used to deliver siRNAs to cancer cells *in vivo*. Optimized siRNAs targeting KRAS impaired colon cancer growth *in vivo* while combinatorial inhibition of KRAS and PIK3CA/PIK3CB significantly improved tumor control compared to single agents alone, demonstrating that targets can be effectively multiplexed (106). In contrast to liposomal or other nanoparticle technologies, exosome-mediated delivery of siRNAs have greater efficiency due to longer persistence in the circulation and take advantage of RAS-mediated upregulation of micropinocytosis for greater uptake by RAS-driven cells. Exosomal delivery of siRNAs targeting KRAS reduced expression of KRAS, suppressed tumor formation, and inhibited metastatic progression in mouse pancreatic cancer models (107).

### Inhibitors of RAS Modulators

Besides directly targeting the enzymatic domain of RAS, many studies have investigated targeting its subcellular localization. As described previously, RAS relies on a number of factors for post-translational modifications and localization to the cell membrane. The phosphodiesterase PDE-delta binds to farnesylated RAS and promotes its efficient signaling by selectively localizing RAS to the plasma membrane as opposed to intracellular membranes (108). Inhibition of the interaction between PDE-delta and KRAS disrupted RAS localization and signaling and impaired cell proliferation in pancreatic cancer models (109). Additionally, inhibition of the lysophospholipase APT1 with palmostatin B blocked RAS depalmitoylation and impaired RAS localization and signaling efficacy and contributed to re-acquisition of contact inhibition in HRAS-transformed fibroblasts (110). This inhibitor demonstrated similar effects in NRAS-driven hematologic cancer models (111). Farnesyltransferases are also essential for RAS membrane localization and represent therapeutic targets. Several of these agents have shown promise in clinical trials by disrupting RAS signaling in combination with other therapeutic agents (112–114), although these effects may be based on inhibition of other farnesylation-dependent enzymes beyond RAS. RAS geranylgeranylation following inhibition of farnesyltransferases reactivates RAS signaling and serves as a common resistance mechanism (115). Combinatorial targeting of farnesyl and geranylgeranyltransferases may overcome this resistance (116).

SOS is a RAS-specific guanine exchange factor (GEF) that mediates the conversion of RAS from an inactive GDP-bound state to an active GTP-bound state. Because of this important role in regulating RAS activity, SOS is a natural target for RAS driven cancers. Helical proteins that interrupt the RAS-SOS interaction blocked RAS activation and downstream ERK activity following EGFR stimulation (117). Additional studies have identified small molecules that can interrupt the RAS-SOS interaction and disrupt RAS activation and downstream MAPK and PI3K signaling (118, 119). In order to mediate its downstream effects, RAS binds to a series of effector molecules through a RAS binding domain. Inhibition of this RAS binding domain with the small molecule agent rigosertib impairs the interaction between RAS and RAF, as well as Ral and PI3K, simultaneously incapacitating several downstream RAS effectors and impairing tumor growth *in vitro* and *in vivo* (120). RAS also relies on kinase suppressor of ras (KSR), which serves as a scaffolding factor that links RAS to RAF and allows for MEK activation (121–124). Stabilization of the inactive form of KSR with small molecule compounds blocked this signal transduction from RAS to RAF and enhanced efficacy of MEK inhibitors (125). STK19 activates oncogenic signaling in melanoma cells through selective phosphorylation of mutant NRAS, which supports its interaction with downstream effectors through the RAS binding domain. Pharmacologic inhibitors of STK19 blocked NRAS phosphorylation and impaired melanoma cell growth and tumor formation capacity, and extended survival of tumor-bearing mice (126).

RAS can also be activated by the protein tyrosine phosphatase SHP2 (encoded by the PTPN11 gene). SHP2 binds to receptor tyrosine kinase growth factor receptors through its SH2 domain and mediates activation of RAS through dephosphorylation of RAS, increasing its association with RAF (127, 128). Inhibition of the SHP2 phosphatase domain with a small molecule inhibitor suppressed RAS signaling and impaired proliferation of receptor tyrosine kinase-driven cancer cells *in vitro* and *in vivo*, although RAS-mutant cells were not sensitive to this drug *in vitro* (129). Targeting SHP2 further sensitized pancreatic cancer cells to MEK inhibition and promoted a senescence response in KRAS-mutant non-small cell lung cancer models under nutrient-restricted conditions (130, 131). These findings suggest that combinatorial targeting of signaling elements upstream and downstream of RAS may be a useful therapeutic approach.

### Inhibition of Downstream Signaling and Resistance Mechanisms

Aberrant RAS activation can also be targeted through inhibition of downstream signaling elements, such as MEK. Despite these efforts, targeted therapies are frequently plagued by the robust emergence of resistance. In KRAS mutant cancers, targeting of MEK with trametinib led to compensatory signaling through fibroblast growth factor receptor 1 (FGFR1). Combinatorial therapy using trametinib and FGFR1 inhibition effectively abolished this resistance mechanism and served as a useful combinatorial strategy (132). RAS-driven cancer cells could further overcome MEK inhibition through overexpression of ERBB3. Targeting the related RTKs EGFR and ERBB2 reversed this effect and sensitized to MEK inhibitors (133). Targeting RAF kinases can also reverse resistance to MEK inhibitors through downregulation of MAPK signaling (134). MEK inhibitors further drive compensatory activating phosphorylation of the KSR-1 scaffolding protein, which promotes PI3K-AKT signaling that circumvents inhibition of RAS signaling effectors (135). In the context of RAF or MEK inhibition, YAP1, a component of the Hippo pathway, promoted survival of RAS-mutant cells, with combinatorial inhibition of MEK and YAP1 yielding improved therapeutic efficacy (136). Thus, development of therapeutic resistance following RAS inhibition is exceedingly common. Greater understanding of these resistance mechanisms may allow researchers to collapse the great degree of cellular plasticity in these signaling networks through combinatorial inhibition of survival and escape pathways.

Despite our detailed understanding of the major RAS downstream signaling elements in cancers, recent evidence revealed that the temporal dynamics of signal transduction, and not just the pathway constituents themselves, are critical to the resulting biological effects. Because of this phenomenon, treatment with BRAF inhibitors may have counterproductive effects on RAS signaling by prolonging the typically short pulses of RAS activity into long periods of downstream ERK activation (137). Furthermore, while BRAF inhibitors are effective in blocking growth of cancer cells driven by the BRAF-V600E mutation, BRAF inhibition paradoxically activates MAPK signaling in KRAS mutant tumors through inducing increased dimerization of BRAF with RAS (138). Because the complexity of these signaling pathways has not been completely elucidated, caution must be used when developing therapeutic agents and their downstream effects must be empirically determined.

### Identification of RAS-Specific Synthetic Lethality

In addition to targeting RAS signaling directly through its enzymatic activity or indirectly through its regulators or downstream signaling effectors, therapeutic targeting of dependencies established by oncogenic RAS is a promising approach. The unique cellular states established by RAS activation create new nodes of fragility that may be amenable to anti-cancer therapies. Increased RAS copy number engages a glycolytic switch which increases glycolysis and shifts glucose utilization toward the TCA cycle and glutathione synthesis. These metabolic changes create sensitivity to glutathione synthesis inhibitors (57). Loss of wild-type RAS further sensitized cells to MEK inhibition, suggesting that allelic imbalance at the KRAS locus can impact dependency on downstream signaling elements (60). Additionally, increased levels of the GLUT1 glucose transporter facilitates selective sensitivity of RAS driven cancers to vitamin C, the oxidized version of which is preferentially imported, depleting intracellular glutathione, and generating oxidative stress (139). Other targeting approaches have leveraged oxidative stress to selectively ablate NF1- or KRAS- mutant tumors through combinatorial therapy with HDAC and mTOR inhibitors, which suppress glutathione synthesis and the thioredoxin antioxidant pathway (140). These findings suggest that RAS driven cancers are particularly vulnerable to oxidative damage and are unable to efficiently cope with oxidative stress. KRAS-driven cancers employ micropinocytosis to scavenge nutrients from the extracellular environment. Through interacting with cell surface integrins, the carbohydrate binding protein galectin-3 mediates formation of macropinosomes and reduces reactive oxygen species by recruiting KRAS clusters on the cell membrane to promote RAS signaling. This event can be effectively targeted with galectin-3 inhibitors (141).

Whole genome shRNA and CRISPR screening strategies have identified RAS-specific synthetic lethalities, elucidating potential novel therapeutic targets. Cells with oncogenic RAS rely on TBK1, an IκB kinase, to activate NF-κB signaling to prevent apoptosis (142). KRAS-driven non-small cell lung cancers also rely on the nuclear export receptor XPO1, which clears nuclear IκBα and supports NF-κB activity. KRAS-mutant models are selectively sensitive to small molecule inhibition of XPO1 (143). RAS mutant non-small cell lung cancers are specifically dependent on GATA2, a transcription factor that regulates the proteasome, Rho signaling pathways, and maintenance of NF-κB signaling via the IL-1 pathway (144). Collectively, these results point toward NF-κB signaling as an essential pro-survival signal selectively utilized by RAS driven cancers. Furthermore, the protein kinase STK33 is a RAS-dependent essential factor that inhibits mitochondrial apoptosis downstream of S6-kinase (S6K1) signaling (145). In the context of MEK inhibition, the mitochondrial anti-apoptotic gene BCL-XL is essential in RAS-driven cancers. Combinatorial inhibition of BCL-XL with MEK signaling enhanced cell death in colorectal, lung, and pancreatic cancers bearing RAS mutations, suggesting that BCL-XL displays a synthetic lethal interaction with RAS in a context-specific manner (146).

Other screens have demonstrated increased dependence on ribosomal biogenesis and translational control, protein neddylation, protein sumoylation, RNA splicing pathways, and mitotic control in RAS mutant cancer models (147). PLK1, a kinase involved in centrosome maturation and spindle assembly during mitotic progression, was specifically essential and targeting this kinase with a small molecule inhibitor selectively targeted RAS mutant cells. This dependence on mitotic machinery and sensitivity to mitotic stress was specific to RAS-mutant cells when compared to PIK3CA driven cells (147). The cell cycle regulator CDK4 also displays a synthetic lethal relationship with KRAS in non-small cell lung cancers (148). A guanine nucleotide exchange factor for Rac family GTPases, PREX1, is essential for MAPK activation in RAS mutant acute myeloid leukemias, and cells driven by oncogenic RAS were sensitized to Rac/PAK family inhibitors (149).

### Immunotherapies

**Figure 6:**
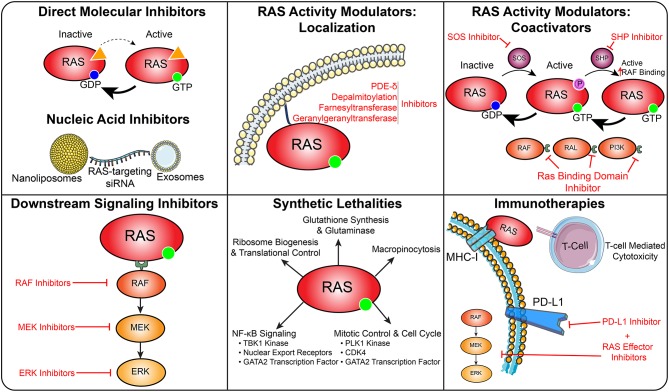
Therapeutic targeting of RAS in cancer. This diagram depicts several strategies to therapeutically target RAS driven cancers.

Therapeutic approaches that harness the immune system to target cancers have emerged as an effective strategy. Recently, CD8+ T-cells have been isolated from a patient with metastatic colorectal cancer that specifically recognize mutant KRAS. *Ex vivo* expansion of this population followed by reinfusion into the patient led to reduction in metastatic burden, suggesting that immunotherapeutic approaches may be effective in targeting RAS (150, 151). Further immunotherapeutic efforts have utilized T-cell receptors engineered to specifically target oncogenic forms of KRAS to control tumor growth in pancreatic cancer models (152) In addition to direct targeting of RAS antigens, immunotherapeutic approaches have been explored in combination with inhibition of downstream RAS signaling elements. In BRAF-driven melanomas, combination of BRAF, MEK, and immune checkpoint inhibition through PD-L1 inhibitors enhanced cancer cell death and displayed efficacy in early clinical trials for metastatic melanoma (153, 154). Combinations of MEK and BRAF inhibitors with PD-L1 inhibitors demonstrated some promise in metastatic colorectal cancers and melanomas in early clinical trials (155, 156). PI3K signaling downstream of RAS controls interactions between cancer cells and the immune microenvironment. While overactive PI3K signaling driven by PTEN mutations reduced T-cell-mediated cytotoxicity, treatment with a PI3Kβ inhibitor enhanced the efficacy of anti-PD1 antibodies in melanoma models (157) (Figure 6).

## Conclusions

RAS family members are some of the most commonly altered genes in cancer. Perturbations of RAS signaling establish robust oncogenic circuits that drive tumor initiation, progression, growth, and survival. Despite our deep knowledge of the direct downstream signaling effectors of the RAS pathway, continued exploration has revealed new insights into the similarities and differences between RAS family members and their preference for particular cancer types. These efforts have also uncovered the more distal downstream consequences of RAS signaling across cancers, including its rewiring of cellular metabolism and capacity to unlock nutrient scavenging pathways, its role in metastasis, and its dual role in regulating the immune microenvironment. These processes endow cancer cells with the plasticity required for survival in dynamic conditions, but also create key vulnerabilities, which can be therapeutically targeted through a number of avenues. Taken together, a deeper understanding of RAS biology will critically inform clinical care and serves as a model for interrogation of other driver alterations in cancer.

## Author Contributions

RG and XW contributed to the conception, research, writing, editing, and design of figures for this manuscript.

### Conflict of Interest

The authors declare that the research was conducted in the absence of any commercial or financial relationships that could be construed as a potential conflict of interest.

## References

[1] Harvey JJ. An unidentified virus which causes the rapid production of tumours in mice. Nature. (1964) 204:1104–5. 10.1038/2041104b0 14243400

[2] Kirsten WH Mayer LA. Morphologic responses to a murine erythroblastosis virus. J Natl Cancer Inst. (1967) 39:311–35. 18623947

[3] Chang EH Gonda MA Ellis RW Scolnick EM Lowy DR. Human genome contains four genes homologous to transforming genes of Harvey and Kirsten murine sarcoma viruses. Proc Natl Acad Sci USA. (1982) 79:4848–52. 10.1073/pnas.79.16.4848 6289320 PMC346782

[4] Ellis RW Defeo D Shih TY Gonda MA Young HA Tsuchida N. The p21 src genes of Harvey and Kirsten sarcoma viruses originate from divergent members of a family of normal vertebrate genes. Nature. (1981) 292:506–11. 10.1038/292506a0 6265801

[5] Parada LF Tabin CJ Shih C Weinberg RA. Human EJ bladder carcinoma oncogene is homologue of Harvey sarcoma virus RAS gene. Nature. (1982) 297:474–8. 10.1038/297474a0 6283357

[6] Santos E Tronick SR Aaronson SA Pulciani S Barbacid M. T24 human bladder carcinoma oncogene is an activated form of the normal human homologue of BALB- and Harvey-MSV transforming genes. Nature. (1982) 298:343–7. 10.1038/298343a0 6283384

[7] Buday L Downward J. Epidermal growth factor regulates p21RAS through the formation of a complex of receptor, Grb2 adapter protein, and Sos nucleotide exchange factor. Cell. (1993) 73:611–20. 10.1016/0092-8674(93)90146-H 8490966

[8] Arvidsson AK Rupp E Nanberg E Downward J Ronnstrand L Wennstrom S. Tyr-716 in the platelet-derived growth factor beta-receptor kinase insert is involved in GRB2 binding and RAS activation. Mol Cell Biol. (1994) 14:6715–26. 10.1128/MCB.14.10.6715 7935391 PMC359202

[9] Bortner DM Ulivi M Roussel MF Ostrowski MC. The carboxy-terminal catalytic domain of the GTPase-activating protein inhibits nuclear signal transduction and morphological transformation mediated by the CSF-1 receptor. Genes Dev. (1991) 5:1777–85. 10.1101/gad.5.10.1777 1717344

[10] Koch WJ Hawes BE Allen LF Lefkowitz RJ. Direct evidence that Gi-coupled receptor stimulation of mitogen-activated protein kinase is mediated by G beta gamma activation of p21ras. Proc Natl Acad Sci USA. (1994) 91:12706–10. 10.1073/pnas.91.26.12706 7809106 PMC45508

[11] Wan Y Kurosaki T Huang XY. Tyrosine kinases in activation of the MAP kinase cascade by G-protein-coupled receptors. Nature. (1996) 380:541–4. 10.1038/380541a0 8606776

[12] Clark EA Hynes RO RAS activation is necessary for integrin-mediated activation of extracellular signal-regulated kinase 2 and cytosolic phospholipase A2 but not for cytoskeletal organization. J Biol Chem. (1996) 271:14814–8. 10.1074/jbc.271.25.14814 8663348

[13] Bos JL Rehmann H Wittinghofer A. GEFs and GAPs: critical elements in the control of small G proteins. Cell. (2007) 129:865–77. 10.1016/j.cell.2007.05.018 17540168

[14] Hancock JF Paterson H Marshall CJ. A polybasic domain or palmitoylation is required in addition to the CAAX motif to localize p21RAS to the plasma membrane. Cell. (1990) 63:133–9. 10.1016/0092-8674(90)90294-O 2208277

[15] Swarthout JT Lobo S Farh L Croke MR Greentree WK Deschenes RJ. DHHC9 and GCP16 constitute a human protein fatty acyltransferase with specificity for H- and N-Ras. J Biol Chem. (2005) 280:31141–8. 10.1074/jbc.M504113200 16000296

[16] Rocks O Gerauer M Vartak N Koch S Huang ZP Pechlivanis M. The palmitoylation machinery is a spatially organizing system for peripheral membrane proteins. Cell. (2010) 141:458–71. 10.1016/j.cell.2010.04.007 20416930

[17] Rocks O Peyker A Kahms M Verveer PJ Koerner C Lumbierres M. An acylation cycle regulates localization and activity of palmitoylated RAS isoforms. Science. (2005) 307:1746–52. 10.1126/science.1105654 15705808

[18] Hancock JF Magee AI Childs JE Marshall CJ. All RAS proteins are polyisoprenylated but only some are palmitoylated. Cell. (1989) 57:1167–77. 10.1016/0092-8674(89)90054-8 2661017

[19] Zhou Y Prakash P Liang H Cho KJ Gorfe AA Hancock JF. Lipid-sorting specificity encoded in K-RAS membrane anchor regulates signal output. Cell. (2017) 168:239–51.e16. 10.1016/j.cell.2016.11.059 28041850 PMC5653213

[20] Ambrogio C Kohler J Zhou ZW Wang H Paranal R Li J. KRAS dimerization impacts MEK inhibitor sensitivity and oncogenic activity of mutant KRAS. Cell. (2018) 172:857–68.e15. 10.1016/j.cell.2017.12.020 29336889

[21] Baker R Wilkerson EM Sumita K Isom DG Sasaki AT Dohlman HG. Differences in the regulation of K-RAS and H-RAS isoforms by monoubiquitination. J Biol Chem. (2013) 288:36856–62. 10.1074/jbc.C113.525691 24247240 PMC3873545

[22] Sasaki AT Carracedo A Locasale JW Anastasiou D Takeuchi K Kahoud ER. Ubiquitination of K-RAS enhances activation and facilitates binding to select downstream effectors. Science Signal. (2011) 4:ra13. 10.1126/scisignal.2001518 21386094 PMC3437993

[23] Yan H Chin ML Horvath EA Kane EA Pfleger CM. Impairment of ubiquitylation by mutation in Drosophila E1 promotes both cell-autonomous and non-cell-autonomous Ras-ERK activation *in vivo*. J Cell Sci. (2009) 122:1461–70. 10.1242/jcs.042267 19366732 PMC2721006

[24] Bigenzahn JW Collu GM Kartnig F Pieraks M Vladimer GI Heinz LX. LZTR1 is a regulator of RAS ubiquitination and signaling. Science. (2018) 362:1171–7. 10.1126/science.aap8210 30442766 PMC6794158

[25] Steklov M Pandolfi S Baietti MF Batiuk A Carai P Najm P. Mutations in LZTR1 drive human disease by dysregu6lating RAS ubiquitination. Science. (2018) 362:1177–82. 10.1126/science.aap7607 30442762 PMC8058620

[26] Yang MH Laurent G Bause AS Spang R German N Haigis MC. HDAC6 and SIRT2 regulate the acetylation state and oncogenic activity of mutant K-RAS. Mol Cancer Res. (2013) 11:1072–7. 10.1158/1541-7786.MCR-13-0040-T 23723075 PMC3778089

[27] Yang MH Nickerson S Kim ET Liot C Laurent G Spang R. Regulation of RAS oncogenicity by acetylation. Proc Natl Acad Sci USA. (2012) 109:10843–8. 10.1073/pnas.1201487109 22711838 PMC3390846

[28] Tan L Cho KJ Kattan WE Garrido CM Zhou Y Neupane P. Acylpeptide hydrolase is a novel regulator of KRAS plasma membrane localization and function. J Cell Sci. (2019) 132:jcs232132. 10.1242/jcs.232132 31266814 PMC6703705

[29] Rajalingam K Schreck R Rapp UR Albert S. RAS oncogenes and their downstream targets. Biochim Biophys Acta. (2007) 1773:1177–95. 10.1016/j.bbamcr.2007.01.012 17428555

[30] Singh A Sweeney MF Yu M Burger A Greninger P Benes C. TAK1 inhibition promotes apoptosis in KRAS-dependent colon cancers. Cell. (2012) 148:639–50. 10.1016/j.cell.2011.12.033 22341439 PMC3291475

[31] Wang MT Holderfield M Galeas J Delrosario R To MD Balmain A. K-RAS promotes tumorigenicity through suppression of non-canonical Wnt signaling. Cell. (2015) 163:1237–51. 10.1016/j.cell.2015.10.041 26590425

[32] Sanchez-Vega F Mina M Armenia J Chatila WK Luna A La KC. Oncogenic signaling pathways in the cancer genome atlas. Cell. (2018) 173:321–37.e10. 10.1016/j.cell.2018.03.035 29625050 PMC6070353

[33] Cerami E Gao J Dogrusoz U Gross BE Sumer SO Aksoy BA. The cBio cancer genomics portal: an open platform for exploring multidimensional cancer genomics data. Cancer Discov. (2012) 2:401–4. 10.1158/2159-8290.CD-12-0095 22588877 PMC3956037

[34] Gao J Aksoy BA Dogrusoz U Dresdner G Gross B Sumer SO. Integrative analysis of complex cancer genomics and clinical profiles using the cBioPortal. Science Signal. (2013) 6:pl1. 10.1126/scisignal.2004088 23550210 PMC4160307

[35] Prior IA Lewis PD Mattos C. A comprehensive survey of RAS mutations in cancer. Cancer Res. (2012) 72:2457–67. 10.1158/0008-5472.CAN-11-2612 22589270 PMC3354961

[36] Biankin AV Waddell N Kassahn KS Gingras MC Muthuswamy LB Johns AL. Pancreatic cancer genomes reveal aberrations in axon guidance pathway genes. Nature. (2012) 491:399–405. 10.1038/nature11547 23103869 PMC3530898

[37] Jones S Zhang X Parsons DW Lin JC Leary RJ Angenendt P. Core signaling pathways in human pancreatic cancers revealed by global genomic analyses. Science. (2008) 321:1801–6. 10.1126/science.1164368 18772397 PMC2848990

[38] Waddell N Pajic M Patch AM Chang DK Kassahn KS Bailey P. Whole genomes redefine the mutational landscape of pancreatic cancer. Nature. (2015) 518:495–501. 10.1038/nature14169 25719666 PMC4523082

[39] Markowitz SD Bertagnolli MM. Molecular origins of cancer: molecular basis of colorectal cancer. N Engl J Med. (2009) 361:2449–60. 10.1056/NEJMra0804588 20018966 PMC2843693

[40] Cancer Genome Atlas Research Network Comprehensive molecular profiling of lung adenocarcinoma. Nature. (2014) 511:543–50. 10.1038/nature13385 25079552 PMC4231481

[41] George J Lim JS Jang SJ Cun Y Ozretic L Kong G. Comprehensive genomic profiles of small cell lung cancer. Nature. (2015) 524:47–53. 10.1038/nature14664 26168399 PMC4861069

[42] Berger MF Hodis E Heffernan TP Deribe YL Lawrence MS Protopopov A. Melanoma genome sequencing reveals frequent PREX2 mutations. Nature. (2012) 485:502–6. 10.1038/nature11071 22622578 PMC3367798

[43] Cancer Genome Atlas Research Network Ley TJ Miller C Ding L Raphael BJ Mungall AJ. Genomic and epigenomic landscapes of adult de novo acute myeloid leukemia. N Engl J Med. (2013) 368:2059–74. 10.1056/NEJMoa1301689 23634996 PMC3767041

[44] Christen F Hoyer K Yoshida K Hou HA Waldhueter N Heuser M. Genomic landscape and clonal evolution of acute myeloid leukemia with t(8;21): an international study on 331 patients. Blood. (2019) 133:1140–51. 10.1182/blood-2018-05-852822 30610028

[45] Cheng CM Li H Gasman S Huang J Schiff R Chang EC. Compartmentalized RAS proteins transform NIH 3T3 cells with different efficiencies. Mol Cell Biol. (2011) 31:983–97. 10.1128/MCB.00137-10 21189290 PMC3067814

[46] Maher J Baker DA Manning M Dibb NJ Roberts IA. Evidence for cell-specific differences in transformation by N-, H- and K-ras. Oncogene. (1995) 11:1639–47. 7478589

[47] Castellano E Santos E. Functional specificity of RAS isoforms: so similar but so different. Genes Cancer. (2011) 2:216–31. 10.1177/1947601911408081 21779495 PMC3128637

[48] Hobbs GA Der CJ Rossman KL. RAS isoforms and mutations in cancer at a glance. J Cell Sci. (2016) 129:1287–92. 10.1242/jcs.182873 26985062 PMC4869631

[49] Nussinov R Tsai CJ Jang H. Oncogenic RAS isoforms signaling specificity at the membrane. Cancer Res. (2018) 78:593–602. 10.1158/0008-5472.CAN-17-2727 29273632 PMC5811325

[50] Yun J Rago C Cheong I Pagliarini R Angenendt P Rajagopalan H. Glucose deprivation contributes to the development of KRAS pathway mutations in tumor cells. Science. (2009) 325:1555–9. 10.1126/science.1174229 19661383 PMC2820374

[51] Gaglio D Metallo CM Gameiro PA Hiller K Danna LS Balestrieri C. Oncogenic K-RAS decouples glucose and glutamine metabolism to support cancer cell growth. Mol Syst Biol. (2011) 7:523. 10.1038/msb.2011.56 21847114 PMC3202795

[52] Ying H Kimmelman AC Lyssiotis CA Hua S Chu GC Fletcher-Sananikone E. Oncogenic KRAS maintains pancreatic tumors through regulation of anabolic glucose metabolism. Cell. (2012) 149:656–70. 10.1016/j.cell.2012.01.058 22541435 PMC3472002

[53] Hutton JE Wang X Zimmerman LJ Slebos RJ Trenary IA Young JD. Oncogenic KRAS and BRAF drive metabolic reprogramming in colorectal cancer. Mol Cell Proteomics. (2016) 15:2924–38. 10.1074/mcp.M116.058925 27340238 PMC5013308

[54] Son J Lyssiotis CA Ying H Wang X Hua S Ligorio M. Glutamine supports pancreatic cancer growth through a KRAS-regulated metabolic pathway. Nature. (2013) 496:101–5. 10.1038/nature12040 23535601 PMC3656466

[55] Romero R Sayin VI Davidson SM Bauer MR Singh SX LeBoeuf SE. Keap1 loss promotes Kras-driven lung cancer and results in dependence on glutaminolysis. Nat Med. (2017) 23:1362–8. 10.1038/nm.4407 28967920 PMC5677540

[56] Santana-Codina N Roeth AA Zhang Y Yang A Mashadova O Asara JM. Oncogenic KRAS supports pancreatic cancer through regulation of nucleotide synthesis. Nature Commun. (2018) 9:4945. 10.1038/s41467-018-07472-8 30470748 PMC6251888

[57] Kerr EM Gaude E Turrell FK Frezza C Martins CP. Mutant KRAS copy number defines metabolic reprogramming and therapeutic susceptibilities. Nature. (2016) 531:110–3. 10.1038/nature16967 26909577 PMC4780242

[58] Weinberg F Hamanaka R Wheaton WW Weinberg S Joseph J Lopez M. Mitochondrial metabolism and ROS generation are essential for Kras-mediated tumorigenicity. Proc Natl Acad Sci USA. (2010) 107:8788–93. 10.1073/pnas.1003428107 20421486 PMC2889315

[59] Mueller S Engleitner T Maresch R Zukowska M Lange S Kaltenbacher T. Evolutionary routes and KRAS dosage define pancreatic cancer phenotypes. Nature. (2018) 554:62–8. 10.1038/nature25459 29364867 PMC6097607

[60] Burgess MR Hwang E Mroue R Bielski CM Wandler AM Huang BJ. KRAS Allelic imbalance enhances fitness and modulates MAP kinase dependence in cancer. Cell. (2017) 168:817–29.e15. 10.1016/j.cell.2017.01.020 28215705 PMC5541948

[61] Bar-Sagi D Feramisco JR. Induction of membrane ruffling and fluid-phase pinocytosis in quiescent fibroblasts by RAS proteins. Science. (1986) 233:1061–8. 10.1126/science.3090687 3090687

[62] Porat-Shliom N Kloog Y Donaldson JG. A unique platform for H-RAS signaling involving clathrin-independent endocytosis. Mol Biol Cell. (2008) 19:765–75. 10.1091/mbc.e07-08-0841 18094044 PMC2262976

[63] Commisso C Davidson SM Soydaner-Azeloglu RG Parker SJ Kamphorst JJ Hackett S. Macropinocytosis of protein is an amino acid supply route in Ras-transformed cells. Nature. (2013) 497:633–7. 10.1038/nature12138 23665962 PMC3810415

[64] Kamphorst JJ Cross JR Fan J de Stanchina E Mathew R White EP. Hypoxic and Ras-transformed cells support growth by scavenging unsaturated fatty acids from lysophospholipids. Proc Natl Acad Sci USA. (2013) 110:8882–7. 10.1073/pnas.1307237110 23671091 PMC3670379

[65] Gwinn DM Lee AG Briones-Martin-Del-Campo M Conn CS Simpson DR Scott AI. Oncogenic KRAS regulates amino acid homeostasis and asparagine biosynthesis via ATF4 and alters sensitivity to L-asparaginase. Cancer Cell. (2018) 33:91–107.e6. 10.1016/j.ccell.2017.12.003 29316436 PMC5761662

[66] Guo JY Chen HY Mathew R Fan J Strohecker AM Karsli-Uzunbas G. Activated RAS requires autophagy to maintain oxidative metabolism and tumorigenesis. Genes Dev. (2011) 25:460–70. 10.1101/gad.2016311 21317241 PMC3049287

[67] Guo JY Teng X Laddha SV Ma S Van Nostrand SC Yang Y. Autophagy provides metabolic substrates to maintain energy charge and nucleotide pools in Ras-driven lung cancer cells. Genes Dev. (2016) 30:1704–17. 10.1101/gad.283416.116 27516533 PMC5002976

[68] Guo JY Karsli-Uzunbas G Mathew R Aisner SC Kamphorst JJ Strohecker AM. Autophagy suppresses progression of K-ras-induced lung tumors to oncocytomas and maintains lipid homeostasis. Genes Dev. (2013) 27:1447–61. 10.1101/gad.219642.113 23824538 PMC3713426

[69] Perera RM Stoykova S Nicolay BN Ross KN Fitamant J Boukhali M. Transcriptional control of autophagy-lysosome function drives pancreatic cancer metabolism. Nature. (2015) 524:361–5. 10.1038/nature14587 26168401 PMC5086585

[70] Padanad MS Konstantinidou G Venkateswaran N Melegari M Rindhe S Mitsche M. Fatty acid oxidation mediated by Acyl-CoA synthetase long chain 3 is required for mutant KRAS lung tumorigenesis. Cell Rep. (2016) 16:1614–28. 10.1016/j.celrep.2016.07.009 27477280 PMC4981512

[71] Mayers JR Torrence ME Danai LV Papagiannakopoulos T Davidson SM Bauer MR. Tissue of origin dictates branched-chain amino acid metabolism in mutant Kras-driven cancers. Science. (2016) 353:1161–5. 10.1126/science.aaf5171 27609895 PMC5245791

[72] Boutin AT Liao WT Wang M Hwang SS Karpinets TV Cheung H. Oncogenic KRAS drives invasion and maintains metastases in colorectal cancer. Genes Dev. (2017) 31:370–82. 10.1101/gad.293449.116 28289141 PMC5358757

[73] Yang K Li Y Lian G Lin H Shang C Zeng L. KRAS promotes tumor metastasis and chemoresistance by repressing RKIP via the MAPK-ERK pathway in pancreatic cancer. In J Cancer. (2018) 142:2323–34. 10.1002/ijc.31248 29315556

[74] Ji H Ramsey MR Hayes DN Fan C McNamara K Kozlowski P. LKB1 modulates lung cancer differentiation and metastasis. Nature. (2007) 448:807–10. 10.1038/nature06030 17676035

[75] Kottakis F Nicolay BN Roumane A Karnik R Gu H Nagle JM. LKB1 loss links serine metabolism to DNA methylation and tumorigenesis. Nature. (2016) 539:390–5. 10.1038/nature20132 27799657 PMC5988435

[76] Schreiber RD Old LJ Smyth MJ. Cancer immunoediting: integrating immunity's roles in cancer suppression and promotion. Science. (2011) 331:1565–70. 10.1126/science.1203486 21436444

[77] Lohmann S Wollscheid U Huber C Seliger B. Multiple levels of MHC class I down-regulation by RAS oncogenes. Scand J Immunol. (1996) 43:537–44. 10.1046/j.1365-3083.1996.d01-73.x 8633212

[78] Seliger B Harders C Wollscheid U Staege MS Reske-Kunz AB Huber C. Suppression of MHC class I antigens in oncogenic transformants: association with decreased recognition by cytotoxic T lymphocytes. Exp Hematol. (1996) 24:1275–9. 8862437

[79] Coelho MA de Carne Trecesson S Rana S Zecchin D Moore C Molina-Arcas M. Oncogenic RAS signaling promotes tumor immunoresistance by stabilizing PD-L1 mRNA. Immunity. (2017) 47:1083–99.e1086. 10.1016/j.immuni.2017.11.016 29246442 PMC5746170

[80] Borghaei H Paz-Ares L Horn L Spigel DR Steins M Ready NE. Nivolumab versus docetaxel in advanced nonsquamous non-small-cell lung cancer. N Engl J Med. (2015) 373:1627–39. 10.1056/NEJMoa1507643 26412456 PMC5705936

[81] Dong ZY Zhong WZ Zhang XC Su J Xie Z Liu SY. Potential predictive value of TP53 and KRAS mutation status for response to PD-1 blockade immunotherapy in lung adenocarcinoma. Clin Cancer Res. (2017) 23:3012–24. 10.1158/1078-0432.CCR-16-2554 28039262

[82] Xu Y Poggio M Jin HY Shi Z Forester CM Wang Y. Translation control of the immune checkpoint in cancer and its therapeutic targeting. Nat Med. (2019) 25:301–11. 10.1038/s41591-018-0321-2 30643286 PMC6613562

[83] Kortlever RM Sodir NM Wilson CH Burkhart DL Pellegrinet L Brown Swigart L. Myc cooperates with RAS by programming inflammation and immune suppression. Cell. (2017) 171:1301–15.e14. 10.1016/j.cell.2017.11.013 29195074 PMC5720393

[84] Caetano MS Zhang H Cumpian AM Gong L Unver N Ostrin EJ. IL6 blockade reprograms the lung tumor microenvironment to limit the development and progression of K-ras-mutant lung cancer. Cancer Res. (2016) 76:3189–99. 10.1158/0008-5472.CAN-15-2840 27197187 PMC4891282

[85] Zhu Z Aref AR Cohoon TJ Barbie TU Imamura Y Yang S. Inhibition of KRAS-driven tumorigenicity by interruption of an autocrine cytokine circuit. Cancer Discov. (2014) 4:452–65. 10.1158/2159-8290.CD-13-0646 24444711 PMC3980023

[86] Ruscetti M Leibold J Bott MJ Fennell M Kulick A Salgado NR. NK cell-mediated cytotoxicity contributes to tumor control by a cytostatic drug combination. Science. (2018) 362:1416–22. 10.1126/science.aas9090 30573629 PMC6711172

[87] Cheng H Fan K Luo G Fan Z Yang C Huang Q Kras(G12D) mutation contributes to regulatory T cell conversion through activation of the MEK/ERK pathway in pancreatic cancer. Cancer Lett. (2019) 446:103–11. 10.1016/j.canlet.2019.01.013 30664964

[88] Schabath MB Welsh EA Fulp WJ Chen L Teer JK Thompson ZJ. Differential association of STK11 and TP53 with KRAS mutation-associated gene expression, proliferation and immune surveillance in lung adenocarcinoma. Oncogene. (2016) 35:3209–16. 10.1038/onc.2015.375 26477306 PMC4837098

[89] McAllister F Bailey JM Alsina J Nirschl CJ Sharma R Fan H. Oncogenic KRAS activates a hematopoietic-to-epithelial IL-17 signaling axis in preinvasive pancreatic neoplasia. Cancer Cell. (2014) 25:621–37. 10.1016/j.ccr.2014.03.014 24823639 PMC4072043

[90] Sparmann A Bar-Sagi D. Ras-induced interleukin-8 expression plays a critical role in tumor growth and angiogenesis. Cancer Cell. (2004) 6:447–58. 10.1016/j.ccr.2004.09.028 15542429

[91] Rak J Mitsuhashi Y Bayko L Filmus J Shirasawa S Sasazuki T. Mutant RAS oncogenes upregulate VEGF/VPF expression: implications for induction and inhibition of tumor angiogenesis. Cancer Res. (1995) 55:4575–80. 7553632

[92] Ji H Houghton AM Mariani TJ Perera S Kim CB Padera R. K-RAS activation generates an inflammatory response in lung tumors. Oncogene. (2006) 25:2105–12. 10.1038/sj.onc.1209237 16288213

[93] Ostrem JM Peters U Sos ML Wells JA Shokat KM. K-Ras(G12C) inhibitors allosterically control GTP affinity and effector interactions. Nature. (2013) 503:548–51. 10.1038/nature12796 24256730 PMC4274051

[94] Lito P Solomon M Li LS Hansen R Rosen N. Allele-specific inhibitors inactivate mutant KRAS G12C by a trapping mechanism. Science. (2016) 351:604–8. 10.1126/science.aad6204 26841430 PMC4955282

[95] Patricelli MP Janes MR Li LS Hansen R Peters U Kessler LV. Selective inhibition of oncogenic KRAS output with small molecules targeting the inactive state. Cancer Discov. (2016) 6:316–29. 10.1158/2159-8290.CD-15-1105 26739882

[96] Janes MR Zhang J Li LS Hansen R Peters U Guo X. Targeting KRAS mutant cancers with a covalent G12C-specific inhibitor. Cell. (2018) 172:578–89.e17. 10.1016/j.cell.2018.01.006 29373830

[97] Gorfe AA Cho KJ Approaches to inhibiting oncogenic K-Ras. Small GTPases. (2019) 10:1–10. 10.1080/21541248.2019.1655883 31438765 PMC7849769

[98] Ni D Li X He X Zhang H Zhang J Lu S Drugging K-Ras(G12C) through covalent inhibitors: mission possible? Pharmacol Ther. (2019) 202:1–17. 10.1016/j.pharmthera.2019.06.007 31233765

[99] Welsch ME Kaplan A Chambers JM Stokes ME Bos PH Zask A. Multivalent small-molecule Pan-RAS inhibitors. Cell. (2017) 168:878–89.e29. 10.1016/j.cell.2017.02.006 28235199 PMC5362268

[100] Lu S Ni D Wang C He X Lin H Wang Z Deactivation pathway of RAS GTPase underlies conformational substates as targets for drug design. ACS Catal. (2019) 9:7188–96. 10.1021/acscatal.9b02556.

[101] McCarthy MJ Pagba C Prakash P Naji AK van der Hoeven D Liang H. Discovery of high-affinity noncovalent allosteric KRAS inhibitors that disrupt effector binding. ACS Omega. (2019) 4:2921–30. 10.1021/acsomega.8b03308 30842983 PMC6396121

[102] Lu S Jang H Gu S Zhang J Nussinov R. Drugging RAS GTPase: a comprehensive mechanistic and signaling structural view. Chem Soc Rev. (2016) 45:4929–52. 10.1039/C5CS00911A 27396271 PMC5021603

[103] Lu S Jang H Muratcioglu S Gursoy A Keskin O Nussinov R. RAS conformational ensembles, allostery, and signaling. Chem Rev. (2016) 116:6607–65. 10.1021/acs.chemrev.5b00542 26815308

[104] Pecot CV Wu SY Bellister S Filant J Rupaimoole R Hisamatsu T. Therapeutic silencing of KRAS using systemically delivered siRNAs. Mol Cancer Ther. (2014) 13:2876–85. 10.1158/1535-7163.MCT-14-0074 25281617 PMC4416486

[105] Seviour EG Sehgal V Mishra D Rupaimoole R Rodriguez-Aguayo C Lopez-Berestein G. Targeting KRas-dependent tumour growth, circulating tumour cells and metastasis *in vivo* by clinically significant miR-193a-3p. Oncogene. (2017) 36:1339–50. 10.1038/onc.2016.308 27669434 PMC5344721

[106] Yuan TL Fellmann C Lee CS Ritchie CD Thapar V Lee LC. Development of siRNA payloads to target KRAS-mutant cancer. Cancer Discov. (2014) 4:1182–97. 10.1158/2159-8290.CD-13-0900 25100204 PMC4184972

[107] Kamerkar S LeBleu VS Sugimoto H Yang S Ruivo CF Melo SA. Exosomes facilitate therapeutic targeting of oncogenic KRAS in pancreatic cancer. Nature. (2017) 546:498–503. 10.1038/nature22341 28607485 PMC5538883

[108] Chandra A Grecco HE Pisupati V Perera D Cassidy L Skoulidis F. The GDI-like solubilizing factor PDEdelta sustains the spatial organization and signalling of RAS family proteins. Nat Cell Biol. (2011) 14:148–58. 10.1038/ncb2394 22179043

[109] Zimmermann G Papke B Ismail S Vartak N Chandra A Hoffmann M. Small molecule inhibition of the KRAS-PDEdelta interaction impairs oncogenic KRAS signalling. Nature. (2013) 497:638–42. 10.1038/nature12205 23698361

[110] Dekker FJ Rocks O Vartak N Menninger S Hedberg C Balamurugan R. Small-molecule inhibition of APT1 affects RAS localization and signaling. Nat Chem Biol. (2010) 6:449–56. 10.1038/nchembio.362 20418879

[111] Xu J Hedberg C Dekker FJ Li Q Haigis KM Hwang E. Inhibiting the palmitoylation/depalmitoylation cycle selectively reduces the growth of hematopoietic cells expressing oncogenic Nras. Blood. (2012) 119:1032–5. 10.1182/blood-2011-06-358960 22144181 PMC3271715

[112] Berndt N Hamilton AD Sebti SM. Targeting protein prenylation for cancer therapy. Nat Rev Cancer. (2011) 11:775–91. 10.1038/nrc3151 22020205 PMC4037130

[113] Siegel-Lakhai WS Crul M Zhang S Sparidans RW Pluim D Howes A. Phase I and pharmacological study of the farnesyltransferase inhibitor tipifarnib (Zarnestra, R115777) in combination with gemcitabine and cisplatin in patients with advanced solid tumours. Br J Cancer. (2005) 93:1222–9. 10.1038/sj.bjc.6602850 16251868 PMC2361514

[114] Sparano JA Moulder S Kazi A Vahdat L Li T Pellegrino C. Targeted inhibition of farnesyltransferase in locally advanced breast cancer: a phase I and II trial of tipifarnib plus dose-dense doxorubicin and cyclophosphamide. J Clin Oncol. (2006) 24:3013–8. 10.1200/JCO.2005.04.9114 16769985

[115] Whyte DB Kirschmeier P Hockenberry TN Nunez-Oliva I James L Catino JJ. K- and N-RAS are geranylgeranylated in cells treated with farnesyl protein transferase inhibitors. J Biol Chem. (1997) 272:14459–64. 10.1074/jbc.272.22.14459 9162087

[116] Kazi A Xiang S Yang H Chen L Kennedy P Ayaz M. Dual farnesyl and geranylgeranyl transferase inhibitor thwarts mutant KRAS-driven patient-derived pancreatic tumors. Clin Cancer Res. (2019). [Epub ahead of print]. 10.1158/1078-0432.CCR-18-3399 31227505 PMC6774803

[117] Patgiri A Yadav KK Arora PS Bar-Sagi D. An orthosteric inhibitor of the Ras-Sos interaction. Nat Chem Biol. (2011) 7:585–7. 10.1038/nchembio.612 21765406 PMC3312813

[118] Burns MC Sun Q Daniels RN Camper D Kennedy JP Phan J. Approach for targeting RAS with small molecules that activate SOS-mediated nucleotide exchange. Proc Natl Acad Sci USA. (2014) 111:3401–6. 10.1073/pnas.1315798111 24550516 PMC3948241

[119] Maurer T Garrenton LS Oh A Pitts K Anderson DJ Skelton NJ. Small-molecule ligands bind to a distinct pocket in RAS and inhibit SOS-mediated nucleotide exchange activity. Proc Natl Acad Sci USA. (2012) 109:5299–304. 10.1073/pnas.1116510109 22431598 PMC3325706

[120] Athuluri-Divakar SK Vasquez-Del Carpio R Dutta K Baker SJ Cosenza SC Basu I. A small molecule RAS-mimetic disrupts RAS association with effector proteins to block signaling. Cell. (2016) 165:643–55. 10.1016/j.cell.2016.03.045 27104980 PMC5006944

[121] Brennan DF Dar AC Hertz NT Chao WC Burlingame AL Shokat KM. A Raf-induced allosteric transition of KSR stimulates phosphorylation of MEK. Nature. (2011) 472:366–9. 10.1038/nature09860 21441910

[122] Rajakulendran T Sahmi M Lefrancois M Sicheri F Therrien M. A dimerization-dependent mechanism drives RAF catalytic activation. Nature. (2009) 461:542–5. 10.1038/nature08314 19727074

[123] Sundaram M Han M. The *C. elegans* ksr-1 gene encodes a novel Raf-related kinase involved in Ras-mediated signal transduction. Cell. (1995) 83:889–901. 10.1016/0092-8674(95)90205-8 8521513

[124] Therrien M Chang HC Solomon NM Karim FD Wassarman DA Rubin GM. KSR, a novel protein kinase required for RAS signal transduction. Cell. (1995) 83:879–88. 10.1016/0092-8674(95)90204-X 8521512

[125] Dhawan NS Scopton AP Dar AC. Small molecule stabilization of the KSR inactive state antagonizes oncogenic RAS signalling. Nature. (2016) 537:112–6. 10.1038/nature19327 27556948 PMC5161575

[126] Yin C Zhu B Zhang T Liu T Chen S Liu Y. Pharmacological targeting of STK19 inhibits oncogenic NRAS-driven melanomagenesis. Cell. (2019) 176:1113–27.e1116. 10.1016/j.cell.2019.01.002 30712867

[127] Bunda S Burrell K Heir P Zeng L Alamsahebpour A Kano Y. Inhibition of SHP2-mediated dephosphorylation of RAS suppresses oncogenesis. Nat Commun. (2015) 6:8859. 10.1038/ncomms9859 26617336 PMC4674766

[128] Matozaki T Murata Y Saito Y Okazawa H Ohnishi H. Protein tyrosine phosphatase SHP-2: a proto-oncogene product that promotes RAS activation. Cancer Sci. (2009) 100:1786–93. 10.1111/j.1349-7006.2009.01257.x 19622105 PMC11158110

[129] Chen YN LaMarche MJ Chan HM Fekkes P Garcia-Fortanet J Acker MG. Allosteric inhibition of SHP2 phosphatase inhibits cancers driven by receptor tyrosine kinases. Nature. (2016) 535:148–52. 10.1038/nature18621 27362227

[130] Mainardi S Mulero-Sanchez A Prahallad A Germano G Bosma A Krimpenfort P. SHP2 is required for growth of KRAS-mutant non-small-cell lung cancer *in vivo*. Nat Med. (2018) 24:961–7. 10.1038/s41591-018-0023-9 29808006

[131] Ruess DA Heynen GJ Ciecielski KJ Ai J Berninger A Kabacaoglu D. Mutant KRAS-driven cancers depend on PTPN11/SHP2 phosphatase. Nat Med. (2018) 24:954–60. 10.1038/s41591-018-0024-8 29808009

[132] Manchado E Weissmueller S Morris JP Chen CC Wullenkord R. A combinatorial strategy for treating KRAS-mutant lung cancer. Nature. (2016) 534:647–51. 10.1038/nature18600 27338794 PMC4939262

[133] Sun C Hobor S Bertotti A Zecchin D Huang S Galimi F. Intrinsic resistance to MEK inhibition in KRAS mutant lung and colon cancer through transcriptional induction of ERBB3. Cell Rep. (2014) 7:86–93. 10.1016/j.celrep.2014.02.045 24685132

[134] Lamba S Russo M Sun C Lazzari L Cancelliere C Grernrum W. RAF suppression synergizes with MEK inhibition in KRAS mutant cancer cells. Cell Rep. (2014) 8:1475–83. 10.1016/j.celrep.2014.07.033 25199829

[135] Kim JY Welsh EA Fang B Bai Y Kinose F Eschrich SA. Phosphoproteomics reveals MAPK inhibitors enhance MET- and EGFR-Driven AKT signaling in KRAS-mutant lung cancer. Mol Cancer Res. (2016) 14:1019–29. 10.1158/1541-7786.MCR-15-0506 27422710 PMC5065770

[136] Lin L Sabnis AJ Chan E Olivas V Cade L Pazarentzos E. The Hippo effector YAP promotes resistance to RAF- and MEK-targeted cancer therapies. Nat Genet. (2015) 47:250–6. 10.1038/ng.3218 25665005 PMC4930244

[137] Bugaj LJ Sabnis AJ Mitchell A Garbarino JE Toettcher JE Bivona TG. Cancer mutations and targeted drugs can disrupt dynamic signal encoding by the Ras-Erk pathway. Science. (2018) 361:eaao3048. 10.1126/science.aao3048 30166458 PMC6430110

[138] Hatzivassiliou G Song K Yen I Brandhuber BJ Anderson DJ Alvarado R. RAF inhibitors prime wild-type RAF to activate the MAPK pathway and enhance growth. Nature. (2010) 464:431–5. 10.1038/nature08833 20130576

[139] Yun J Mullarky E Lu C Bosch KN Kavalier A Rivera K. Vitamin C selectively kills KRAS and BRAF mutant colorectal cancer cells by targeting GAPDH. Science. (2015) 350:1391–6. 10.1126/science.aaa5004 26541605 PMC4778961

[140] Malone CF Emerson C Ingraham R Barbosa W Guerra S Yoon H. mTOR and HDAC inhibitors converge on the TXNIP/thioredoxin pathway to cause catastrophic oxidative stress and regression of RAS-driven tumors. Cancer Discov. (2017) 7:1450–63. 10.1158/2159-8290.CD-17-0177 28963352 PMC5718976

[141] Seguin L Camargo MF Wettersten HI Kato S Desgrosellier JS von Schalscha T. Galectin-3, a druggable vulnerability for KRAS-addicted cancers. Cancer Discov. (2017) 7:1464–79. 10.1158/2159-8290.CD-17-0539 28893801 PMC5718959

[142] Barbie DA Tamayo P Boehm JS Kim SY Moody SE Dunn IF. Systematic RNA interference reveals that oncogenic KRAS-driven cancers require TBK1. Nature. (2009) 462:108–12. 10.1038/nature08460 19847166 PMC2783335

[143] Kim J McMillan E Kim HS Venkateswaran N Makkar G Rodriguez-Canales J. XPO1-dependent nuclear export is a druggable vulnerability in KRAS-mutant lung cancer. Nature. (2016) 538:114–7. 10.1038/nature19771 27680702 PMC5161658

[144] Kumar MS Hancock DC Molina-Arcas M Steckel M East P Diefenbacher M. The GATA2 transcriptional network is requisite for RAS oncogene-driven non-small cell lung cancer. Cell. (2012) 149:642–55. 10.1016/j.cell.2012.02.059 22541434

[145] Scholl C Frohling S Dunn IF Schinzel AC Barbie DA Kim SY. Synthetic lethal interaction between oncogenic KRAS dependency and STK33 suppression in human cancer cells. Cell. (2009) 137:821–34. 10.1016/j.cell.2009.03.017 19490892

[146] Corcoran RB Cheng KA Hata AN Faber AC Ebi H Coffee EM. Synthetic lethal interaction of combined BCL-XL and MEK inhibition promotes tumor regressions in KRAS mutant cancer models. Cancer Cell. (2013) 23:121–8. 10.1016/j.ccr.2012.11.007 23245996 PMC3667614

[147] Luo J Emanuele MJ Li D Creighton CJ Schlabach MR Westbrook TF. A genome-wide RNAi screen identifies multiple synthetic lethal interactions with the RAS oncogene. Cell. (2009) 137:835–48. 10.1016/j.cell.2009.05.006 19490893 PMC2768667

[148] Puyol M Martin A Dubus P Mulero F Pizcueta P Khan G. A synthetic lethal interaction between K-Ras oncogenes and Cdk4 unveils a therapeutic strategy for non-small cell lung carcinoma. Cancer Cell. (2010) 18:63–73. 10.1016/j.ccr.2010.05.025 20609353

[149] Wang T Yu H Hughes NW Liu B Kendirli A Klein K. Gene essentiality profiling reveals gene networks and synthetic lethal interactions with oncogenic Ras. Cell. (2017) 168:890–903.e15. 10.1016/j.cell.2017.01.013 28162770 PMC5445660

[150] Tran E Ahmadzadeh M Lu YC Gros A Turcotte S Robbins PF. Immunogenicity of somatic mutations in human gastrointestinal cancers. Science. (2015) 350:1387–90. 10.1126/science.aad1253 26516200 PMC7445892

[151] Tran E Robbins PF Lu YC Prickett TD Gartner JJ Jia L. T-cell transfer therapy targeting mutant KRAS in cancer. N Engl J Med. (2016) 375:2255–62. 10.1056/NEJMoa1609279 27959684 PMC5178827

[152] Wang QJ Yu Z Griffith K Hanada K Restifo NP Yang JC. Identification of T-cell receptors targeting KRAS-mutated human tumors. Cancer Immunol Res. (2016) 4:204–14. 10.1158/2326-6066.CIR-15-0188 26701267 PMC4775432

[153] Hu-Lieskovan S Mok S Homet Moreno B Tsoi J Robert L Goedert L. Improved antitumor activity of immunotherapy with BRAF and MEK inhibitors in BRAF(V600E) melanoma. Sci Transl Med. (2015) 7:279ra241. 10.1126/scitranslmed.aaa4691 25787767 PMC4765379

[154] Ribas A Lawrence D Atkinson V Agarwal S Miller WH Jr Carlino MS Combined BRAF and MEK inhibition with PD-1 blockade immunotherapy in BRAF-mutant melanoma. Nat Med. (2019) 25:936–40. 10.1038/s41591-019-0476-5 31171879 PMC8562134

[155] Hellmann MD Kim TW Lee CB Goh BC Miller WH Oh DY. Phase Ib study of atezolizumab combined with cobimetinib in patients with solid tumors. Ann Oncol. (2019) 30:1134–42. 10.1093/annonc/mdz113 30918950 PMC6931236

[156] Sullivan RJ Hamid O Gonzalez R Infante JR Patel MR Hodi FS. Atezolizumab plus cobimetinib and vemurafenib in BRAF-mutated melanoma patients. Nat Med. (2019) 25:929–35. 10.1038/s41591-019-0474-7 31171876

[157] Peng W Chen JQ Liu C Malu S Creasy C Tetzlaff MT. Loss of PTEN promotes resistance to T cell-mediated immunotherapy. Cancer Discov. (2016) 6:202–16. 10.1158/2159-8290.CD-15-0283 26645196 PMC4744499

